# Supplementary material for: Supplementation with High or Low Iron Reduces Colitis Severity in an AOM/DSS Mouse Model
Source: Nutrients. 2022 May 12;14(10):2033. doi: 10.3390/nu14102033 (PMC9147005; doi:10.3390/nu14102033)
Supplement: Supplementary file 1 [file nutrients-14-02033-s001.zip › nutrients-1694847-supplementary.pdf]

- **Supplementary Figure S1.**

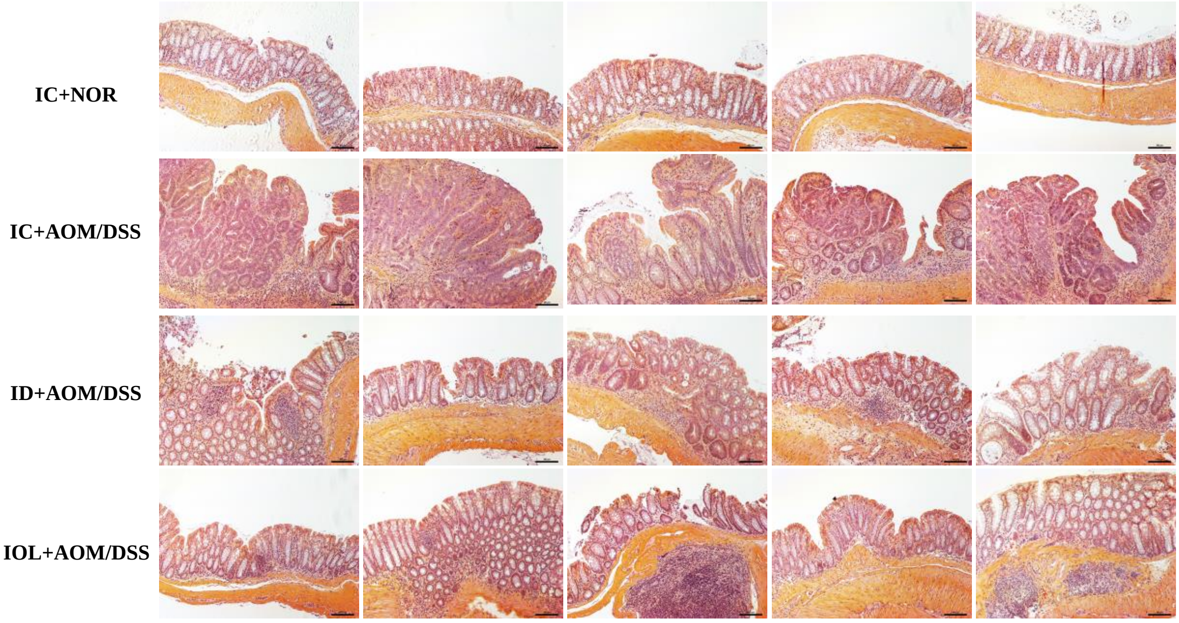

H&E staining of the large intestine tissues. Histologic examination showed the presence of larger adenomas and increased areas of inflammatory cell infiltration in colonic sections from AOM/DSS-treated mice. Scale bar = 100 μm. Abbreviations: NOR, normal; AOM/DSS, azoxymethane/dextran sodium sulfate; IC, iron control; ID, iron-deficient; IOL, iron overload.

- **Supplementary Figure S2.**

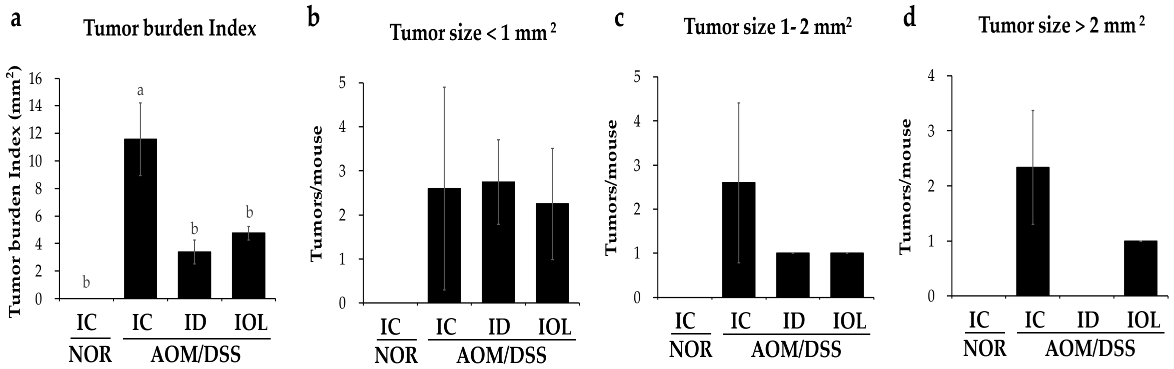

Tumor burden index and tumor size. Tumor areas in the colon of each group were added up and presented as a tumor burden index (a). Average number of tumors in each group by tumor size; < $1mm^2$ (b), 1-2 mm^2 (c), > 2 mm^2 (d). The data are presented as mean ± SD. One-way ANOVA was used for the test of difference and Tukey for the post-hoc test. Different letters above the value indicate statistical significance. Abbreviations: NOR, normal; AOM/DSS, azoxymethane/dextran sodium sulfate; IC, iron control; ID, iron-deficient; IOL, iron overload.

- **Supplementary Table S1.**

Groups		IC+NOR	IC+AOM/DSS	ID+AOM/DSS	IOL+AOM/DSS
n		5	8	6	8
Body weight (g)		35.0 ± 2.52[a]	28.4 ± 2.29[ab]	20.6 ± 2.84[b]	33.3 ± 9.99[a]
Relative tissue Weight (g/kg body weight)	Liver	38.6 ± 3.21[b]	44.0 ± 3.4[b]	54.7 ± 1.42[a]	42.1 ± 9.21[b]
	Small intestine	85.3 ± 6.08[b]	109.7 ± 10[a]	27.3 ± 2.88[c]	23.9 ± 4.82[c]
	Large intestine	15.9 ± 2.01[bc]	23.5 ± 6.77[ab]	24.5 ± 5.76[a]	12.6 ± 4.02[c]
	Spleen	3.87 ± 0.86[b]	5.80 ± 2.89[b]	15.8 ± 11.6[a]	2.94 ± 0.69[b]
	Kidney	11.2 ± 1.07[b]	10.9 ± 0.61[b]	13.6 ± 0.90[a]	10.6 ± 2.36[b]

Body and organ weights of mice fed different iron-concentrated diets a-d Means with different letters in the column are significantly different ($p < 0.05$) according to Tukey's multiple range test. Mean ± SD. Abbreviations: NOR, normal; AOM/DSS, azoxymethane/dextran sodium sulfate; IC, iron control; ID, iron-deficient; IOL, iron overload.
